# Submucosal tunneling endoscopic fenestration resection combined with a covered stent for a large leiomyoma of the esophagogastric junction

**DOI:** 10.1055/a-2589-0938

**Published:** 2025-05-19

**Authors:** Xueyi Lin, Min Lin

**Affiliations:** 1 Department of Gastroenterology, The Affiliated Changzhou No. 2 Peopleʼs Hospital of Nanjing Medical University, Changzhou, China

Endoscopic resection of large esophageal leiomyomas extending from the lower esophagus to the gastric cardia is technically challenging 1 , with risks of perforation and subcutaneous emphysema. Traditionally, surgical resection has been the standard treatment 2 . However, advancements in endoscopic techniques have enabled successful treatment of similar tumors 3 .

We present the case of a 53-year-old man who underwent submucosal tunneling endoscopic resection (STER) for a large esophageal leiomyoma, followed by fenestration extraction and placement of a covered stent for defect repair ( Video 1 ). Preoperative computed tomography (CT) revealed irregular thickening and luminal narrowing suggestive of a tumor, which was confirmed by mini-probe endoscopic ultrasonography showing a hypoechoic mass originating from the muscularis propria ( Fig. 1 **a, b** ).

Submucosal tunneling endoscopic fenestration resection combined with a covered stent for a large leiomyoma of the esophagogastric junction.Video 1

**Fig. 1:**
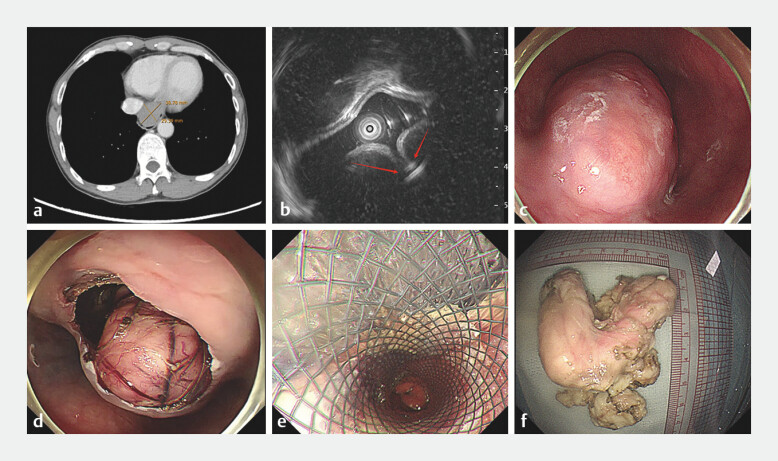
**a** Computed tomography imaging revealed irregular thickening of lower esophagus. **b** Mini-probe endoscopic ultrasound image showing a hypoechoic mass originating from the muscularis propria. **c** Endoscopic image showing a large leiomyoma of the esophagogastric junction. **d** Resection of the tumor along the tunnel. **e** Defect closure by the covered stent. **f** The resected tumor measured 7.0 × 5.0 cm.

Submucosal injection and tunnel creation were performed 36 cm from the incisors, followed by tumor resection along the tunnel ( Fig. 1 **c, d** ). The tumor, originating from the muscularis mucosae and extending toward the serosa near the cardia, was irregular with visible branches. Due to the complexity of complete resection using standard STER, a tunnel window was created for tumor removal. The 7.0 × 5.0-cm tumor could not be extracted intact endoscopically, so it was resected in pieces using a snare and HookKnife (KD-620LR; Olympus, Tokyo, Japan) and removed with a basket. A covered stent (MTN-SE-S-18/60-A-8/650; Micro-tech, Nanjing, China) was placed and secured with clips at the oral end ( Fig. 1 **e, f** ). Three weeks later, endoscopy showed a well-healed resection site, and the stent was removed.

STER is an effective approach for treating non-fusion, elongated tumors in the lower esophagus, preserving mucosal integrity and reducing incision healing time 4 . However, for complex tumor shapes assessed preoperatively, endoscopic full-thickness resection (EFTR) should be considered. In this case, preoperative CT and ultrasound did not adequately assess the tumor's shape and size. Future studies should focus on integrating 3D ultrasound and CT reconstructions to enhance preoperative evaluation and optimize surgical approach selection.

Endoscopy_UCTN_Code_TTT_1AO_2AG_3AB

## Notes

Endoscopy E-Videos https://eref.thieme.de/e-videos
*E-Videos* is an open access online section of the journal *Endoscopy* , reporting on interesting cases and new techniques in gastroenterological endoscopy. All papers include a high-quality video and are published with a Creative Commons CC-BY license. Endoscopy E-Videos qualify for HINARI discounts and waivers and eligibility is automatically checked during the submission process. We grant 100% waivers to articles whose corresponding authors are based in Group A countries and 50% waivers to those who are based in Group B countries as classified by Research4Life (see: https://www.research4life.org/access/eligibility/ ). This section has its own submission website at https://mc.manuscriptcentral.com/e-videos .
